# Linking Seasonal Dietary Strategies and Selectivity to Inform Forage Restoration for Przewalski’s Gazelle on the Qinghai–Tibet Plateau

**DOI:** 10.3390/ani16050794

**Published:** 2026-03-04

**Authors:** Lili Hou, Ming Xu

**Affiliations:** 1 Faculty of Geographic Science and Engineering, College of Geographical Sciences, Henan University, Zhengzhou 450046, China; houlili@henu.edu.cn; 2 Guangdong-Hong Kong Joint Laboratory for Carbon Neutrality, Jiangmen Laboratory of Carbon Science and Technology, Jiangmen 529199, China

**Keywords:** dietary selectivity, Jacobs’ index, forage restoration, seasonal bottleneck, Przewalski’s gazelle

## Abstract

Understanding the forage resources that sustain endangered herbivores under strong seasonal constraints is essential for effective habitat restoration. Przewalski’s gazelle (*Procapra przewalskii*), an endemic ungulate restricted to the Qinghai Lake Basin on the Qinghai–Tibet Plateau, persists in fragmented subpopulations facing pronounced seasonal bottlenecks in forage availability. Here, we investigated seasonal dietary strategies and forage selectivity across nine geographically isolated subpopulations by integrating fecal microhistological diet analysis with vegetation surveys and availability-corrected Jacobs’ electivity indices. Gazelle diets were compressed in early spring, dominated by graminoids (Poaceae and Cyperaceae), but expanded substantially during summer, with increased contributions from Fabaceae and Rosaceae and significantly higher richness and niche breadth. Electivity analyses revealed a hierarchical spectrum of preferences structured around core foundation taxa consistently selected across seasons, complemented by season-specific priority resources during spring bottlenecks and summer abundance. Basin-wide pairwise ranking further identified seasonal priority forage taxa with varying spatial consistency across subpopulations. These findings provide a seasonally explicit framework for identifying key forage targets and guiding evidence-based restoration and zoned management within Qinghai Lake National Park, offering transferable insights for conserving endangered plateau herbivores under fragmentation and strong seasonal resource limitation.

## Simple Summary

The Przewalski’s gazelle is an endangered antelope found only in the Qinghai Lake Basin of China. These animals live in fragmented habitats and face serious challenges in finding enough food, especially during the food-scarce spring. To support effective conservation, it is important to understand not only what plants gazelles eat, but which plants they actively prefer. In this study, we examined the diets of nine gazelle subpopulations across different seasons by comparing plant remains in feces with the vegetation available in their habitats. We found that gazelles depend heavily on a small number of grass species to survive the difficult spring period, but shift to a more diverse diet that includes legumes and high-quality plants during summer. Importantly, gazelles do not simply consume the most abundant plants; they consistently select certain preferred species depending on the season. We identified key forage plants that are essential for gazelle survival. These findings provide practical guidance for habitat restoration and management in Qinghai Lake National Park and contribute to the long-term conservation of endangered herbivores living in seasonally constrained environments.

## 1. Introduction

Herbivores are experiencing widespread global declines [1], with large-bodied ungulates particularly vulnerable to habitat fragmentation, climate change [2,3] and human persecution [4]. These pressures are amplified in high-elevation grasslands characterized by strong seasonality, such as the Qinghai–Tibet Plateau [5]. Przewalski’s gazelle (*Procapra przewalskii*), an endemic and endangered ungulate restricted to the Qinghai Lake Basin, now persists in several geographically isolated subpopulations and plays an important role in maintaining alpine grassland ecosystem structure [6,7]. Despite recent conservation progress, the species continues to face severe threats from ongoing habitat fragmentation and human disturbance [8]. Understanding the ecological basis of its foraging strategies is therefore essential for developing effective management measures [9,10], particularly in the context of the newly established Qinghai Lake National Park [11].

Food resources form the primary link between herbivores and their environment, directly governing survival and reproduction [12]. Dietary ecology thus provides critical insights into how ungulates meet nutritional demands under fluctuating environmental conditions [13,14]. However, many studies remain descriptive, reporting diet composition without simultaneously quantifying plant availability [13,15,16]. This limitation makes it difficult to distinguish active selection from passive consumption based on local abundance [14]. Assessing dietary choices relative to environmental resource availability provides a robust conceptual framework to quantify true forage selectivity and its seasonal shifts [17]. Such selectivity-based approaches are widely used across ungulate systems to identify priority forage taxa, offering valuable guidance for habitat restoration and conservation management [18,19].

The application of availability-corrected selectivity frameworks to understand the foraging preferences of Przewalski’s gazelle remains limited [20,21]. Existing studies have largely concentrated on diet composition or habitat use during the summer growing season and are often restricted to single local subpopulations [21]. Although several dominant forage species, such as *Stipa* and *Leymus*, have been identified, these findings rarely integrate dietary intake with quantitative measures of plant availability [20,21]. Consequently, it remains unclear which forage taxa are consistently preferred across the fragmented landscape and which plant resources are most critical during seasonal bottlenecks. This knowledge gap limits the ability to translate dietary information into science-based forage restoration priorities for regional conservation programs.

Here, we investigated the seasonal dietary strategies and forage selectivity of Przewalski’s gazelle across nine geographically isolated subpopulations in the Qinghai Lake Basin. By linking diet composition with environmental resource availability, we quantified true forage selectivity during both the spring bottleneck (April) and the summer growing season (July). Specifically, we aimed to: (i) characterize seasonal shifts in diet composition, diversity, and niche breadth; (ii) identify forage taxa that are actively selected disproportionately to their availability; and (iii) derive season-specific and core priority plant lists to inform forage restoration and zoned management within Qinghai Lake National Park.

## 2. Materials and Methods

### 2.1. Study Area

**Figure 1:**
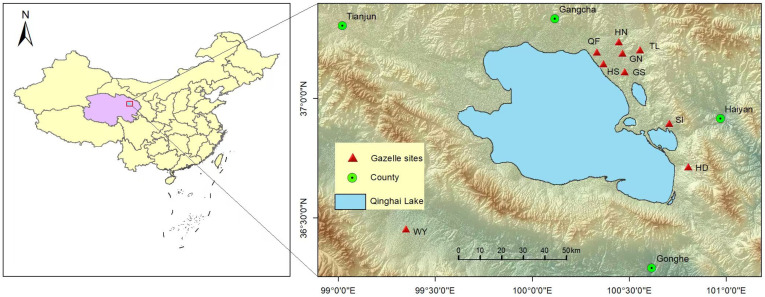
Locations of the nine geographically isolated Przewalski’s gazelle subpopulations sampled in the Qinghai Lake Basin on the northeastern Qinghai–Tibet Plateau. Qinghai Lake Farm No. 1 Branch Factory (QF), south of Hargai Railway (HS), north of Hargai Railway (HN), Talexuanguo (TL), north of Ganzihe Railway (GN), south of Ganzihe Railway (GS), Sand Island (SI), Hudong (HD), and Wayu (WY).

Our study was conducted in the Qinghai Lake Basin on the northeastern Qinghai–Tibet Plateau (97°05′–101°02′ E, 35°05′–37°05′ N). The region is characterized by a continental plateau climate, with annual mean temperatures ranging from −0.8 °C to 1.1 °C and annual precipitation between 327 and 423 mm [22]. Vegetation is dominated by alpine grasslands and meadows, with common species including *Kobresia humilis*, *Achnatherum splendens*, and *Stipa purpurea* [23]. We selected nine geographically isolated subpopulations of Przewalski’s gazelle (QF, TL, GN, GS, HN, HS, HD, SI, WY) from the core groups consistently covered by our long-term monitoring program in the Qinghai Lake Basin. Together, they broadly represent the species’ remaining distribution and habitat variation, while avoiding areas where taxonomic identification may be complicated by hybridization with sympatric gazelle species (Figure 1).

### 2.2. Vegetation Monitoring and Fecal Sample Collection

Field surveys were conducted in April (spring nutritional bottleneck) and July (summer growing season) of 2023 across all nine gazelle subpopulations. Plant availability was quantified during the peak growing season (July) using quadrat sampling. At each site, at least 10 quadrats (1 × 1 m) were randomly placed along transects spaced approximately 50 m apart within representative habitat patches. Within each quadrat, all vascular plant species were recorded, and their percent cover, height, frequency, and density were measured following standard alpine grassland survey protocols [24,25]. On the Qinghai–Tibet Plateau, the effective plant growing season is short (approximately 3–4 months), during which most annual aboveground biomass and plant taxonomic expression occurs [26]. Moreover, local alpine grasslands are dominated by perennial species (e.g., *Stipa* spp., *Kobresia* spp.) that contribute a stable standing vegetation structure across the year. We therefore used July surveys as a basin-wide baseline of the annual standing vegetation assemblage for availability-corrected analyses. In April, vegetation consisted largely of standing dead biomass and early regreening was limited, which precluded a complete quadrat-based assessment of spring availability; accordingly, spring selectivity patterns are interpreted conservatively as bottleneck-stage resource reliance relative to the annual vegetation background [27].

Fecal samples were collected concurrently in both seasons. At each subpopulation, fresh dry pellets were gathered from multiple locations to ensure representative sampling. Pellets were distinguished from sympatric livestock dung based on morphology and associated field signs [14]. All samples were geo-referenced and stored dry prior to laboratory analysis.

### 2.3. Fecal Microhistological Analysis

Diet composition was determined using fecal microhistological analysis following a modified sodium hypochlorite digestion method [28]. Reference slides were prepared from locally collected plant specimens to facilitate fragment identification (Table S1). For each subpopulation and season, fecal pellets were pooled into a composite sample to characterize subpopulation-level dietary patterns. Composite samples were examined under a Leica DM2500 microscope (Leica Microsystems, Wetzlar, Germany) at 100× magnification. Plant epidermal fragments were identified by comparison with the reference collection, and the relative density (RD) of each plant species was calculated to represent its proportional contribution to the diet [14]. To ensure adequate sampling coverage, 300 microscope fields were analyzed per composite sample in April and 400 fields per composite sample in July, reflecting higher plant diversity during the growing season [29]. In fecal microhistological analysis, observer bias often arises from the subjective identification of partially digested plant fragments and the morphological similarity of epidermal structures among closely related plant taxa (e.g., grasses and sedges). Each field was reviewed systematically, and all identifications were conducted by the same trained observer to minimize inter-observer variability and ensure consistency in taxonomic assignment across all samples.

### 2.4. Calculation of Vegetation Importance Values (IV)

Plant importance values (*IV*) were calculated for July quadrats as the mean of relative coverage, relative density, and relative frequency for each species [30]. Specifically, *IV* was computed as

(1) IV=(RC+RD+RF)/3

where RC, RD, and RF represent the relative coverage, relative density, and relative frequency of each species, respectively. These metrics were obtained from field surveys, and *IV* were used to assess the relative availability of each plant species in the gazelle’s foraging habitat.

### 2.5. Diversity and Niche Breadth Indices

Dietary diversity and niche breadth were assessed using the Shannon–Wiener diversity index (*H*), Pielou’s evenness index (*J*), species richness, and Levins’ niche breadth index (*B*), all based on the relative proportion of each plant species in the diet [14,21].

(2) $$H=-\sum_{i=1}^{S}p_{i}\ln p_{i}$$

(3) J=H/lnS

(4) $$B=1/\sum_{i=1}^{S}p_{i}^{2}$$

where *p*_*i*_ is the relative proportion of plant species *i* in the diet, and *S* is the total number of species.

### 2.6. Dietary Selectivity Index

Dietary selectivity was quantified using Jacobs’ index (*D*). This index was chosen for its ability to account for both plant availability and proportional dietary intake, offering a robust measure of preference independent of overall plant abundance [18]:

(5) D=(r−p)/(r+p−2rp)

where *r* is the relative proportion of a plant species in the diet and *p* is its relative proportion in the available vegetation (importance value). Given the constraints of early-season vegetation surveys, July importance values *(IV*) were used as the proxy for *p* in both April and July calculations. For each season, basin-wide priority forage taxa were identified using a cross-subpopulation pairwise comparison ranking approach based on Jacobs’ electivity values. Within each subpopulation, a plant taxon received a “win” when its electivity exceeded that of another taxon; wins were summed across pairwise comparisons and subpopulations to generate seasonal rankings [31]. Median electivity values (median D) and the number of subpopulations with available estimates (n) were also reported to summarize preference strength and spatial consistency. To reduce instability associated with rare taxa and extreme electivity values, electivity analyses were restricted to forage taxa that reached ≥3% in dietary proportion in at least one subpopulation within a given season, and that also occurred in the vegetation dataset [32].

### 2.7. Statistical Analysis

Seasonal differences in dietary diversity indices (species richness, Shannon diversity, Pielou’s evenness, and Levins’ niche breadth) were evaluated using paired *t*-tests, treating each gazelle subpopulation as a matched replicate sampled in both April and July [14]. Prior to analysis, data distributions were checked for normality (Shapiro–Wilk test), and all indices met the assumptions required for parametric paired comparisons. Statistical significance was set at *p* < 0.05. All statistical analyses and figure preparation were conducted in OriginPro 2024, with additional visualization performed in R version 4.4.1.

## 3. Results

### 3.1. Seasonal Variation in Dietary Functional Groups

**Figure 2:**
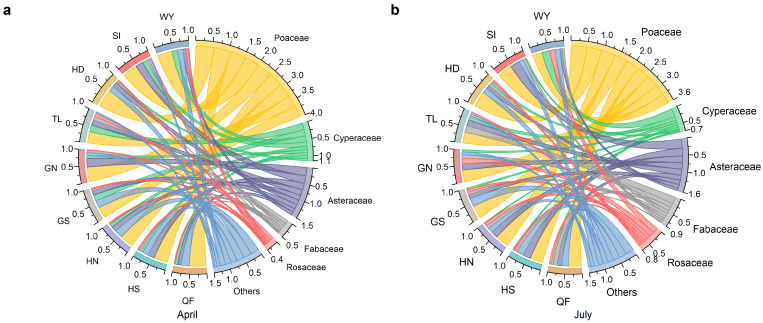
Seasonal changes in the proportional contribution (0–1) of major plant functional groups in the diet of Przewalski’s gazelle across nine subpopulations, determined by fecal analysis. (**a**) April (spring bottleneck) and (**b**) July (summer growing season). Poaceae, Cyperaceae, and Asteraceae dominated early-spring diets, whereas Fabaceae and Rosaceae increased substantially during summer. Colors are used to distinguish the nine Przewalski’s gazelle subpopulations (left) and the major plant families (right), and the ribbons are colored to match the corresponding plant family sectors.

Przewalski’s gazelle exhibited distinct seasonal shifts in the composition of major plant functional groups (Figure 2). In April, the diet was dominated by graminoids (Poaceae and Cyperaceae) and Asteraceae across most subpopulations. By July, the contribution of Fabaceae and Rosaceae increased markedly, accompanied by a relative decline in Poaceae and Cyperaceae, reflecting a seasonal transition toward more diverse herbaceous forages during the growing season. At the subpopulation level, functional-group composition showed spatial variation. In April, Cyperaceae contributed more substantially to the diet in TL (20.5%), GS (19.0%), and WY (18.1%) than in other subpopulations, whereas Asteraceae accounted for a higher proportion in GN (25.9%), SI (23.4%), and HS (20.3%). In July, Fabaceae became more prominent in TL (20.9%), while Rosaceae was relatively more important in WY (16.5%) and GN (15.6%). These results demonstrate both a general increase in dietary diversity during summer and marked spatial heterogeneity in foraging strategies among fragmented subpopulations (Table S2).

### 3.2. Seasonal Variation in Dietary Diversity and Niche Breadth

**Figure 3:**
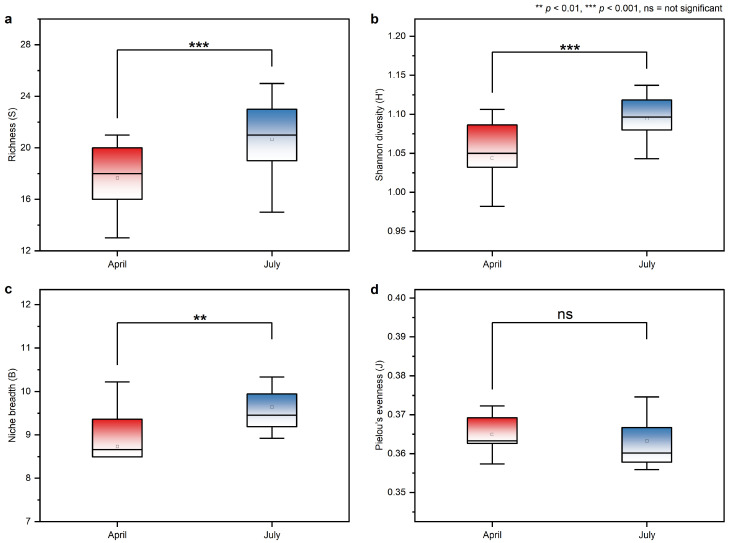
Seasonal variation in dietary diversity indices of Przewalski’s gazelle based on fecal analysis. (**a**) Species richness(*S*); (**b**) Shannon–Wiener diversity index (*H*); (**c**) Levins’ niche breadth index (*B*); and (**d**) Pielou’s evenness index (*J*). Red boxplots represent April and blue boxplots represent July. Boxes indicate the median (line), mean (square), and interquartile range (IQR); whiskers extend to 1.5 × IQR. Significance levels are indicated as: ** *p* < 0.01, and *** *p* < 0.001; “ns” = not significant.

Dietary diversity and niche breadth varied significantly between seasons (Figure 3). Species richness (*S*), Shannon diversity (*H*), and Levins’ niche breadth (*B*) were all significantly higher in July compared to April (Paired *t*-test, *p* < 0.001). This increase is consistent with a broader and more diverse diet during the growing season. In contrast, Pielou’s evenness (*J*) showed no significant seasonal difference (*p* > 0.05), indicating that although more plant species were consumed in summer, the proportional distribution among them remained relatively stable. These patterns reflect seasonal constraints on forage availability, where the spring bottleneck restricts diets to fewer dominant taxa compared to the broader niche supported by the summer growing season.

### 3.3. Vegetation Composition and Availability

Key forage resources were identified by including all plant species with an importance value (*IV*) > 0.05 in at least one subpopulation, together with several habitat-specialist shrubs of ecological relevance, such as *Hippophae tibetana* and *Ephedra monosperma*. Based on July vegetation surveys, regional forage availability was dominated by graminoids, particularly Poaceae and Cyperaceae, with additional contributions from forbs in Asteraceae and Rosaceae (Table 1). These major functional groups were present across all sites, but the relative importance of individual species differed among subpopulations. For example, within Poaceae, *Poa pratensis* showed relatively high importance values in QF (*IV* = 0.179), HN (0.133), and GN (0.125), whereas *Orinus kokonorica* was particularly important in HD (0.311), SI (0.250), and WY (0.110). Among Cyperaceae, *Carex arcatica* had comparatively high values in WY (0.212) and TL (0.153). In Asteraceae, *Artemisia frigida* was more important in GS (0.095), TL (0.090), and HD (0.076). These patterns reveal clear spatial heterogeneity in species-level forage composition among fragmented subpopulations (Table S3).

### 3.4. Dietary Selectivity and Preference Patterns

**Figure 4:**
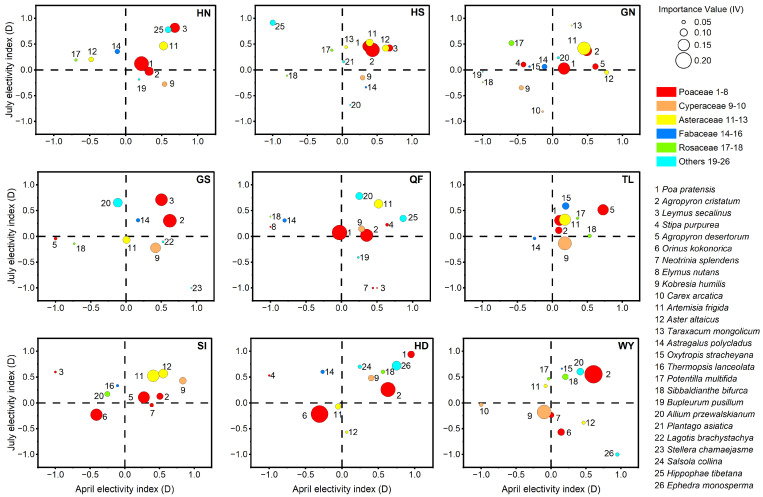
Seasonal dynamics of forage selectivity across nine Przewalski’s gazelle subpopulations. The plots display Jacobs’ electivity index (*D*) for April (*x*-axis) versus July (*y*-axis). Bubble size represents the July Importance Value (*IV*), indicating resource availability. Colors denote functional groups: Poaceae (red), Cyperaceae (orange), Asteraceae (yellow), Fabaceae (blue), Rosaceae (green), and Others (cyan). Numbers correspond to species listed on the right.

Dietary selectivity quantified using Jacobs’ electivity index (*D*) indicated that gazelle foraging was frequently decoupled from local plant availability (Figure 4). Across subpopulations, several locally abundant taxa (large bubbles) showed neutral-to-negative electivity, indicating that high availability did not necessarily translate into high use (e.g., *Carex* spp. in multiple sites). Conversely, a number of preferred taxa exhibited relatively low availability (small bubbles) yet positive electivity, suggesting active selection for high-quality resources despite their scarcity.

Selectivity patterns also differed among functional groups and seasons. Graminoids such as *Agropyron cristatum* showed consistently positive electivity across most subpopulations, indicating stable year-round preference. *Artemisia frigida* (Asteraceae) likewise exhibited broadly positive electivity in both seasons. In contrast, Fabaceae displayed a marked seasonal signal, shifting from neutral or negative electivity in April to strong positive selection in July.

### 3.5. Seasonal Priority Forage Taxa Across the Basin

The ranking analysis revealed distinct seasonal turnover between April and July (Table 2). A subset of taxa consistently ranked highly in both seasons and were therefore classified as core forage resources supported across subpopulations, most notably *Agropyron cristatum* and *Artemisia frigida*. In addition, several taxa emerged as strongly season-specific priorities: *Kobresia humilis* was characteristic of the spring bottleneck selection, whereas *Thermopsis lanceolata* and *Astragalus polycladus* were preferred primarily during the summer growing season. A small number of taxa, such as *Hippophae tibetana*, exhibited high electivity but occurred in relatively few subpopulations, indicating that they function as locally important resources. Together, these seasonal rankings provide a basin-wide, seasonally resolved priority list for guiding forage restoration and species selection in Qinghai Lake National Park (Table S4).

## 4. Discussion

Our study moves beyond descriptive diet lists and biomass-centered assessments by explicitly linking gazelle foraging decisions to resource availability across spatially isolated subpopulations. By integrating diet composition with availability-corrected selectivity across nine subpopulations, we demonstrate that Przewalski’s gazelle foraging is structured around a hierarchical set of consistently selected core taxa, supplemented by season-specific resources. This framework provides both mechanistic insight into dietary strategies and an applied basis for identifying priority forage taxa relevant to management in the Qinghai Lake Basin.

### 4.1. Regional-Scale Patterns of Foraging Strategies

Przewalski’s gazelle exhibited pronounced seasonal shifts in dietary composition and niche breadth, reflecting an intermediate mixed feeder strategy that enables flexible resource use in highly seasonal alpine environments [33,34]. Such seasonal shifts represent a form of phenological tracking, whereby foraging decisions align with temporal changes in plant availability and nutritional quality [35,36]. Importantly, these seasonal patterns varied spatially, with some subpopulations relying heavily on sedges while others shifted more strongly toward forbs, reflecting substantial dietary plasticity in response to local plant assemblages. We interpret this dietary strategy as an adaptive mechanism that may contribute to population persistence in the fragmented and heterogeneous landscapes of the Qinghai Lake Basin.

### 4.2. Core Foundation Taxa and Season-Specific Priority Resources

Availability-corrected selectivity analyses further revealed that gazelle foraging is not structured around a fixed species list, but rather around a hierarchical spectrum of preferences. A shared set of core taxa, primarily dominant graminoids together with widespread forbs such as *Artemisia*, were consistently preferred across subpopulations and seasons, forming a basin-wide forage foundation. These core species are likely favored because they provide reliable baseline energy intake under variable conditions: dominant Poaceae combine high standing biomass and broad spatial coverage with relatively high digestible carbohydrate supply, making them predictable forage resources supporting daily maintenance [37].

Beyond this core set, several taxa emerged as strongly season-specific. Spring priority species captured resources repeatedly selected during the bottleneck period, when regreening is delayed and accessible forage is limited. Increased reliance on sedges and cushion plants such as *Kobresia* spp. likely reflects their persistence as available biomass when other herbaceous resources are scarce [38]. In contrast, summer priority rankings were dominated by legumes and diverse forbs, consistent with selection for nutrient-rich forage during the growing season when plant diversity peaks [13,14].

The observed preference hierarchy further suggests that gazelles balance multiple nutritional objectives beyond energy acquisition alone. Legumes such as *Astragalus* spp. and *Thermopsis lanceolata* may provide critical protein supplementation [39,40], while consistent selection of aromatic taxa such as *Artemisia frigida* and *Allium przewalskianum* is consistent with the use of plants rich in secondary metabolites that could influence gut function or parasite regulation [41,42]. However, high electivity does not necessarily indicate desirable restoration targets. For example, positive electivity for *Stellera chamaejasme* in the HS, GS, QF, and WY subpopulations likely reflects context-dependent foraging under extreme resource scarcity, given its known toxicity [43]. Together, these patterns indicate that gazelle persistence depends jointly on forage quantity, composition, and functional diversity, rather than on biomass availability alone.

### 4.3. Implications for Restoration and Management

Identifying basin-wide core taxa together with season-specific priority resources provides an operational framework for forage restoration in Qinghai Lake National Park. Habitat restoration for Przewalski’s gazelle should prioritize the re-establishment of functionally diverse perennial graminoid communities dominated by *Agropyron, Leymus*, and *Poa*, rather than focusing solely on increasing forage biomass [38]. Emphasizing this diverse guild of grasses promotes community stability while providing a reliable energetic baseline across seasons [44,45].

Supplementary incorporation of native legumes and high-quality forbs (e.g., *Allium przewalskianum*) can further enhance seasonal nutritional carrying capacity by increasing protein availability during the growing season [39,46,47]. Locally selected shrubs such as *Ephedra monosperma* and *Hippophae* spp. are consistently chosen by specific subpopulations, underscoring their conservation value and the need to preserve them where they naturally occur [48,49]. For spring bottleneck habitats, particularly *Kobresia*-dominated meadows, management should emphasize reducing livestock competition [21] and protecting turf integrity during early spring rather than relying on active planting, given the difficulty of restoring turf-forming sedges [50]. Finally, elevated reliance on toxic or unpalatable plants should be interpreted as a warning signal of habitat degradation rather than a conservation objective [51]; high selectivity for species such as *Stellera chamaejasme* reflects constrained forage choice under scarcity and underscores the need for broader range rehabilitation to restore a balanced graminoid–forb community.

Several limitations should be acknowledged. Vegetation availability was quantified in July, whereas early-spring plant availability could not be fully surveyed due to widespread senescence, requiring spring selectivity to be interpreted relative to the broader plant pool. In addition, dietary estimates were derived from composite fecal samples per subpopulation and season, providing robust subpopulation-level patterns but limiting inference on individual variation. Future work combining multi-season vegetation monitoring, individual-based sampling, and complementary approaches such as DNA metabarcoding or tracking could further refine our understanding of dietary adaptation in alpine herbivores.

## 5. Conclusions

This study demonstrates that Przewalski’s gazelle adopts a flexible and seasonally structured foraging strategy to cope with the strong seasonal constraints of the Qinghai–Tibet Plateau. By accounting for plant availability, we showed that graminoids form the dietary foundation throughout the year, whereas the selective use of forbs, particularly Fabaceae and Rosaceae, becomes increasingly important during summer. This seasonal shift likely supports recovery from the severe nutritional limitation experienced in early spring, which remains a key bottleneck for population persistence in fragmented habitats.

Our results provide practical guidance for habitat restoration within Qinghai Lake National Park. Rather than relying on generalized re-greening efforts, restoration strategies should focus on functional forage by prioritizing the reseeding of identified core and seasonally important plant taxa, especially those that alleviate spring food shortages. This targeted approach offers a transferable framework for restoring habitats and supporting population recovery of endangered large herbivores facing strong seasonality and habitat fragmentation in alpine ecosystems.

## Figures and Tables

**Table 1:** Importance values (*IV*) of major and habitat-specific forage taxa recorded in July vegetation quadrat surveys across nine Przewalski’s gazelle subpopulations in the Qinghai Lake Basin.

Family	Species	HN	HS	GN	GS	QF	TL	SI	HD	WY
Poaceae	*Agropyron cristatum*	0.078	0.079	0.052	0.061	0.107	0.069	0.040	0.070	0.069
	*Poa pratensis*	0.133	0.062	0.125	0.080	0.179	0.087	0.025	0.003	0.009
	*Agropyron desertorum*	0.001	0.044	0.029	0.083	0.010	0.032	0.101	0.005	0.035
	*Neotrinia splendens*	0.017	0.041	0.077	0.014	0.013	0.046	0.007	0.015	0.016
	*Stipa purpurea*	0.002	0.023	0.004	0.013	0.021	0.016	0.032	0.012	0.086
	*Elymus nutans*	0.040	0.074	-	0.003	0.032	-	-	-	-
	*Orinus kokonorica*	-	-	-	0.006	-	-	0.250	0.311	0.110
Cyperaceae	*Carex arcatica*	0.033	0.048	0.122	0.088	0.051	0.153	0.015	0.029	0.212
	*Kobresia humilis*	0.056	0.010	0.060	0.040	0.009	0.043	0.012	0.039	0.097
Asteraceae	*Aster altaicus*	0.068	0.026	0.013	0.049	0.107	0.003	0.034	0.051	0.029
	*Artemisia frigida*	0.038	0.034	0.069	0.095	0.037	0.090	0.057	0.076	0.032
	*Artemisia waltonii*	0.016	0.048	0.014	0.002	0.031	0.043	0.014	0.026	0.005
	*Artemisia salsoloides*	-	-	-	-	-	-	0.026	0.104	-
Fabaceae	*Astragalus polycladus*	0.041	0.022	0.069	0.030	0.047	0.051	0.012	0.021	0.005
Rosaceae	*Argentina anserina*	0.033	0.060	-	0.003	0.013	-	0.002	-	-
	*Sibbaldianthe adpressa*	0.013	0.008	0.040	0.075	0.009	0.015	0.002	0.012	0.093
	*Sibbaldianthe bifurca*	0.012	0.042	0.050	0.057	0.016	0.023	0.003	0.012	0.033
	*Potentilla multifida*	0.041	0.022	0.042	0.015	0.029	0.017	-	-	0.022
	*Allium przewalskianum*	0.010	0.025	0.021	0.046	0.020	0.003	0.066	-	0.029
Others	*Lagotis brachystachya*	0.011	0.048	0.001	0.014	0.009	0.008	-	0.002	-
	*Androsace mariae*	0.023	0.046	0.033	-	0.030	-	-	-	-
	*Salsola collina Pall*	-	-	-	0.006	-	-	0.102	0.013	-
	*Stellera chamaejasme*	0.019	0.001	0.008	0.001	0.001	0.030	-	0.033	0.007
	*Ephedra monosperma*	-	-	-	-	-	-	-	0.018	0.003
	*Hippophae tibetana*	0.014	0.007	-	-	0.011	-	-	-	-

“-” indicates that the plant species was absent from the habitat of the corresponding subpopulation.

**Table 2:** Seasonal ranking of the top 15 priority forage taxa for Przewalski’s gazelle across the Qinghai Lake Basin based on availability-corrected dietary selectivity.

Plant	April			July			Categories
	n	Median D	Rank	n	Median D	Rank	
*Agropyron cristatum*	9	0.4892	1	9	0.262965	4	Core
*Aster altaicus*	9	0.4646	2	9	−0.04698	11	Core
*Artemisia frigida*	9	0.3898	3	9	0.418654	1	Core
*Kobresia humilis*	9	0.291	4	9	−0.15035	15	Spring-specific
*Leymus secalinus*	4	0.5905	5	4	0.657147	8	Core
*Thermopsis lanceolata*	6	0.25965	6	7	0.334988	2	Summer-specific
*Allium przewalskianum*	7	0.1141	7	7	0.270824	5	Summer-specific
*Stellera chamaejasme*	4	0.48395	8	1	−0.75267	26	Indicator species
*Poa pratensis*	7	0.1619	9	6	0.217588	10	Core
*Astragalus polycladus*	9	−0.1173	10	9	0.313621	3	Summer-specific
*Neotrinia splendens*	5	0.3868	11	4	−0.13899	21	Spring-specific
*Agropyron desertorum*	4	0.4433	12	6	0.086579	12	Spring-specific
*Sibbaldianthe bifurca*	6	0.21325	13	8	0.124433	9	Summer-specific
*Taraxacum mongolicum*	4	0.163	14	5	0.704576	6	Summer-specific
*Hippophae tibetana*	2	0.7281	15	3	0.72438	14	Local
*Oxytropis stracheyana*	5	0.0592	16	7	0.064692	13	Summer-specific
*Potentilla multifida*	7	−0.1521	18	7	0.353171	7	Summer-specific

n: number of subpopulations where the taxon was recorded. Median D: Jacobs’ electivity index calculated across subpopulations. Values range from −1 (avoidance) to +1 (preference), with 0 indicating random selection.

## Data Availability

The data presented in this study are available on request from the corresponding author.

## Supplementary Materials

The following supporting information can be downloaded at https://www.mdpi.com/article/10.3390/ani16050794/s1, Table S1 Comparison of epidermal micro-characteristics between reference plant tissues and fecal residues; Table S2: Seasonal dietary proportions of major plants in Przewalski’s gazelle (April and July); Table S3: Importance values (*IV*) of plants in nine Przewalski’s gazelle subpopulations’ habitats in July; Table S4: Seasonal ranking of priority forage taxa for Przewalski’s gazelle across the Qinghai Lake Basin based on availability-corrected dietary selectivity.
