# Supplementary material for: Linking Seasonal Dietary Strategies and Selectivity to Inform Forage Restoration for Przewalski’s Gazelle on the Qinghai–Tibet Plateau
Source: Animals (Basel). 2026 Mar 4;16(5):794. doi: 10.3390/ani16050794 (PMC12984604; doi:10.3390/ani16050794)
Supplement: Supplementary file 1 [file animals-16-00794-s001.zip › animals-4166942-supplementary.pdf]

Table S1 Comparison of epidermal micro-characteristics between reference plant tissues and fecal residues.

Family	Plant species	Microscopic view (plants)	Microscopic view (feces)
Poaceae	*Elymus nutans*	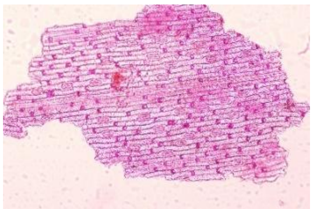	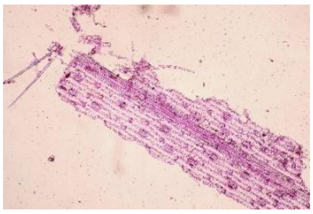
	Agropyron cristatum	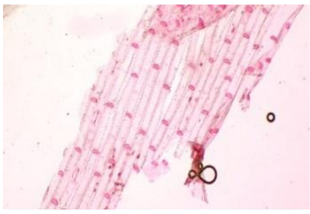	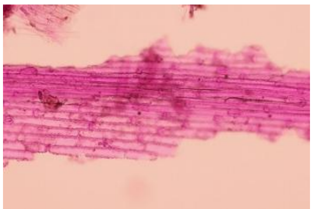
Cyperaceae	*Kobresia humilis*	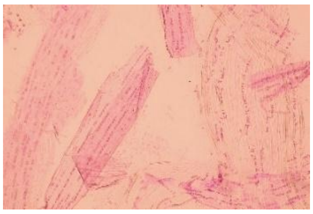	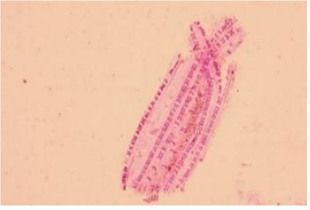
	Carex orbicularis	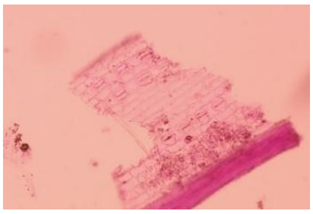	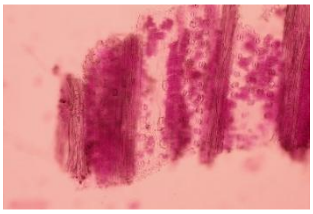
Asteraceae	*Aster altaicus*	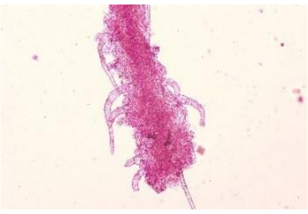	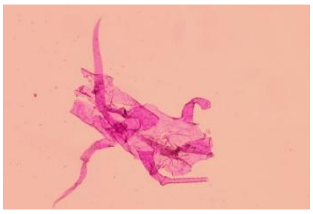
	Artemisia waltonii	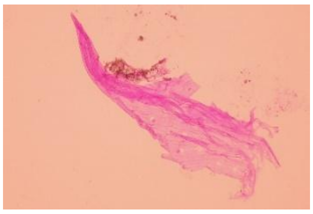	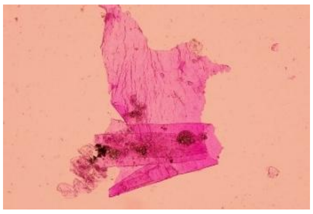
Fabaceae	*Astragalus polycladus*	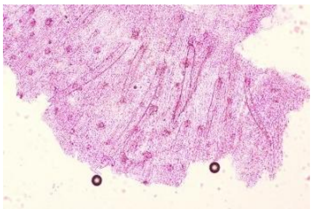	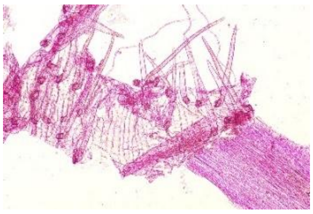

**Family**	**Plant species**	**Microscopic view (plants)**	**Microscopic view (feces)**
	Oxytropis stracheyana	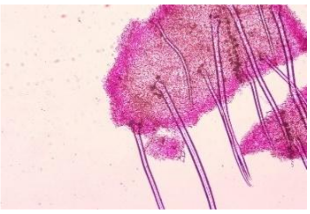	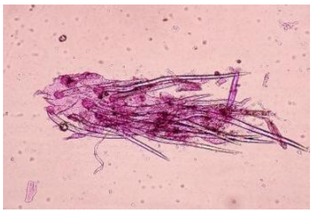
Rosaceae	*Potentilla multifida*	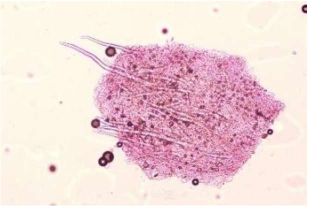	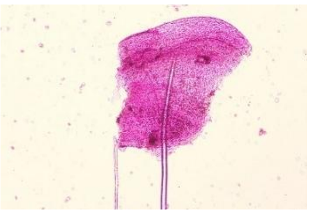
	Sibbaldianthe bifurca	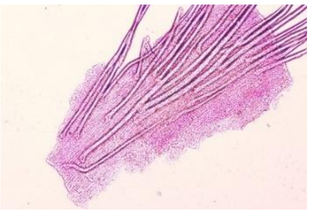	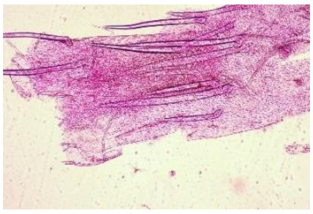
Others	*Hippophae tibetana*	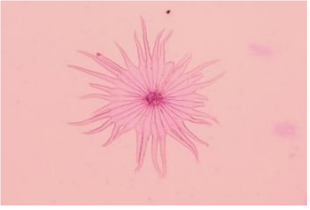	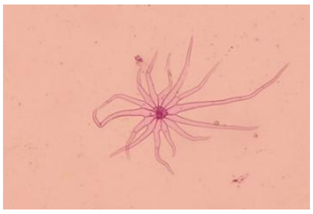
	Allium przewalskianum	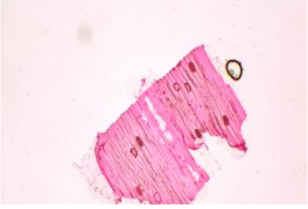	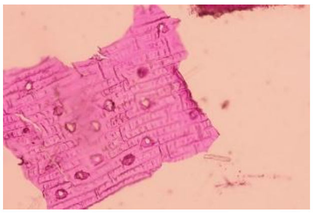
	Stellera chamaejasme	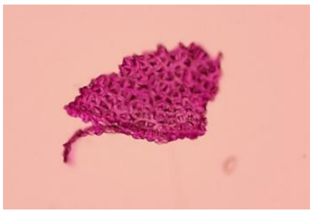	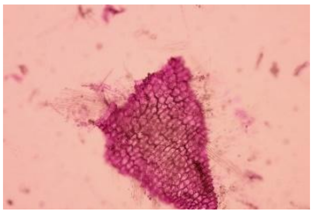
	Ephedra monosperma	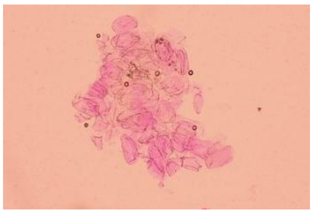	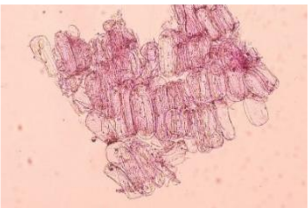

Table S2 Seasonal dietary proportions of major plants in Przewalski's gazelle (April and July)

Plant species and family	RD (%)																	
	**HN**		**HS**		**GN**		**GS**		**QF**		**TL**		**SI**		**HD**		**WY**	
	April	July	April	July	April	July	April	July	April	July	April	July	April	July	April	July	April	July
Poa pratensis	19.25	16.42	12.67	15.22	16.47	13.11	2.73	–	17.08	20.23	10.5	15.42	–	–	9.24	8.19	–	–
Elymus nutans	–	2.18	1.83	2.57	–	–	–	–	–	4.54	–	–	–	–	–	–	–	–
Leymus secalinus	8.84	15.52	10.67	5.4	–	–	10.77	19.25	3.33	–	–	9.37	–	6.33	–	–	–	–
Agropyron desertorum	–	–	–	–	11.07	3.3	–	7.69	1.07	2.02	17.1	–	16.48	12.25	–	0.57	–	–
Agropyron cristatum	14.24	7.45	17.85	16.45	13.84	10.68	21.73	10.9	20.02	11.11	8.21	8.64	11.11	5.08	24.99	12	23.15	20.74
Neotrinia splendens	–	–	–	–	1.24	–	–	2.31	5.33	–	–	0.58	6.89	2.91	2.46	–	8.63	5.5
Orinus kokonorica	–	–	–	–	–	–	–	–	–	–	–	–	12.26	17.35	19.27	22.56	14.21	3.33
Stipa purpurea Griseb	–	0.79	–	–	3.32	9.42	–	–	5.6	2.02	–	–	–	1.66	–	5.14	–	–
**Poaceae**	**42.33**	**42.37**	**43.02**	**39.65**	**45.94**	**36.51**	**35.23**	**40.14**	**52.43**	**39.91**	**35.81**	**34.01**	**46.74**	**45.59**	**55.96**	**48.46**	**45.99**	**29.58**
Kobresia humilis	10.19	1.89	8.33	3.56	4.98	6.31	18.99	5.77	8.66	6.66	20.48	12.11	13.99	3.58	6.47	8.19	18.12	15.95
Carex arcatica	–	0.7	–	–	4.56	0.68	–	–	–	–	–	–	–	–	–	–	–	9.13
**Cyperaceae**	**10.19**	**2.58**	**8.33**	**3.56**	**9.54**	**6.99**	**18.99**	**5.77**	**8.66**	**6.66**	**20.48**	**12.11**	**13.99**	**3.58**	**6.47**	**8.19**	**18.12**	**25.08**
Artemisia frigida	11.15	9.74	7.5	10.43	16.33	15.25	9.67	8.46	9.2	12.43	12.38	16.29	12.65	16.43	6.93	7.05	2.78	6.23
Aster altaicus	2.5	9.94	10.5	6.26	9.13	1.16	1.64	2.56	1.6	2.52	2.15	2.74	10.73	11.33	5.7	1.52	7.52	1.3
Taraxacum mongolicum	0.96	0.79	2.33	5.27	0.41	3.1	2.37	–	–	–	–	–	–	1.08	–	–	–	–
Leontopodium nanum	1.15	–	–	–	–	–	–	–	–	–	2.82	–	–	–	–	–	–	–
Artemisia waltonii	–	–	–	0.98	–	–	–	2.31	–	–	–	–	–	–	–	2.28	–	–
Ajania tenuifolia	–	–	–	0.74	–	–	–	–	–	–	–	–	–	–	–	–	–	–
Artemisia salsoloides	–	–	–	–	–	–	–	–	–	–	–	–	–	–	2.15	0.76	–	–

Plant species and family	RD (%)																	
	**HN**		**HS**		**GN**		**GS**		**QF**		**TL**		**SI**		**HD**		**WY**	
	April	July	April	July	April	July	April	July	April	July	April	July	April	July	April	July	April	July
**Asteraceae**	**15.76**	**20.48**	**20.33**	**23.68**	**25.87**	**19.52**	**13.68**	**13.33**	**10.8**	**14.95**	**17.35**	**19.03**	**23.38**	**28.85**	**14.78**	**11.61**	**10.3**	**7.53**
Astragalus polycladus	3.27	8.35	4.33	1.1	5.53	7.77	4.19	5.64	0.53	8.58	3.09	4.75	2.49	1.08	1.23	8.38	1.11	2.61
Oxytropis stracheyana	–	–	1.67	0.49	1.66	3.69	–	0.38	–	1.72	4.84	11.96	0.77	1.66	–	–	0.97	3.48
Thermopsis lanceolata	0.58	1.29	–	–	0.69	0.78	1.09	2.18	1.73	1.61	1.48	2.88	2.11	5.08	–	1.33	–	–
Oxytropis falcata	–	–	–	–	–	0.48	–	–	–	–	1.75	1.3	1.91	–	–	–	–	–
Medicago archiducis-nicolai	0.58	–	–	–	–	–	–	–	–	–	–	–	–	–	–	–	–	–
**Fabaceae**	**3.85**	**9.64**	**6**	**1.59**	**7.88**	**12.71**	**5.28**	**8.2**	**2.26**	**11.91**	**11.16**	**20.89**	**7.28**	**7.83**	**1.23**	**9.71**	**2.08**	**6.08**
Potentilla multifida	0.77	5.96	1.67	4.91	1.11	12.14	2.55	1.92	1.07	2.42	3.5	3.46	–	–	–	–	2.09	5.94
Sibbaldianthe bifurca	1.92	1.99	0.5	3.43		3.1	0.91	4.36		3.63	6.99	2.3	–	–	4.31	5.14	4.87	9.42
Sibbaldianthe adpressa	–	–	–	0.37	0.28	0.39	–	–	–	–	–	–	–	–	–	–	–	–
Argentina anserina	–	1.59	1.17	1.47	–	–	–	1.02	–	–	–	–	1.72	–	–	–	–	–
**Rosaceae**	**2.69**	**9.54**	**3.34**	**10.18**	**1.39**	**15.63**	**3.46**	**7.3**	**1.07**	**6.05**	**10.49**	**5.76**	**1.72**		**4.31**	**5.14**	**6.96**	**16.52**
Pedicularis kansuensis	0.38	0.79	–	1.35	–	–	–	–	–	–	–	–	–	–	–	–	–	–
Pedicularis alaschanica	–	–	–	–	–	0.29		0.26	–	–	1.61	1.15		1.33	1.54	0.95	–	–
Lagotis brachystachya	–	–	2.17	–	–	–	4.38	1.15	–	–	–	–	–	–	–	1.14	–	–
Lancea tibetica	–	–	–	–	–	–	–	–	–	0.71	–	–	–	–	–	–	–	–
**Scrophulariaceae**	**0.38**	**0.79**	**2.17**	**1.35**		**0.29**	**4.38**	**1.41**		**0.71**	**1.61**	**1.15**		**1.33**	**1.54**	**2.09**		
Bupleurum pusillum	3.27	1.59	–	–	–	3.3	2.01	1.28	1.01	1.66	–	1.58	–	–	–	–	–	–
Carum carvi	–	0.2	–	–	–	–	0.55	–	–	–	–	–	–	–	–	–	–	–
**Apiaceae**	**3.27**	**1.79**	–	–	–	**3.3**	**2.56**	**1.28**	**1.01**	**1.66**	**0**	**1.58**	–	–	–	–	–	–
Dracocephalum heterophyllum	0.58	–	0.5	0.49	2.35	3.4	1.82	18.73	–	14.65	0.4	0.58	–	9.08	0.77	–	–	–

Plant species and family	RD (%)																	
	HN		HS		GN		GS		QF		TL		SI		HD		WY	
	April	July	April	July	April	July	April	July	April	July	April	July	April	July	April	July	April	July
**Labiatae**	**0.58**	–	**0.5**	**0.49**	**2.35**	**3.4**	**1.82**	**18.73**	–	**14.65**	**0.4**	**0.58**	–	**9.08**	**0.77**	–	–	–
Gentiana dahurica	1.54	0.79	–	–	–	–	–	1.54	2.4	1.01	–	–	–	–	–	–	–	–
**Gentianaceae**	**1.54**	**0.79**	–	–	–	–	–	**1.54**	**2.4**	**1.01**	–	–	–	–	–	–	–	–
Lepidium apetalum	–	0.5	0.67	0.37	–	–	–	–	–	–	–	–	–	–	–	–	–	–
Braya humilis	2.88	0.3	–	–	2.07	0.29	0.55	0.26	1.07	0.2	–	0.58	1.34	0.58	1.23	–	–	–
**Cruciferae**	**2.88**	**0.79**	**0.67**	**0.37**	**2.07**	**0.29**	**0.55**	**0.26**	**1.07**	**0.2**	–	**0.58**	**1.34**	**0.58**	**1.23**	–	–	–
Allium przewalskianum	–	–	3.16	2.41	2.49	2.27	3.65	7.82	3.33	1.9	2.02	–	4.02	1.57	–	–	6.68	10.87
**Liliaceae**	–	–	**3.16**	**2.41**	**2.49**	**2.27**	**3.65**	**7.82**	**3.33**	**1.9**	**2.02**	–	**4.02**	**1.57**	–	–	**6.68**	**10.87**
Hippophae tibetana	5.19	10.34	–	13.63	–	–	–	–	13.87	2.32	–	–	–	–	–	–	–	
**Elaeagnaceae**	**5.19**	**10.34**	–	**13.63**	–	–	–	–	**13.87**	**2.32**	–	–	–	–	–	–	–	
Plantago depressa	0.58	–	2.5	3.31	–	–	–	–	–	–	–	–	–	–	–	–	–	2.03
**Plantaginaceae**	**0.58**	–	**2.5**	**3.31**	–	–	–	–	–	–	–	–	–	–	–	–	–	**2.03**
Chenopodium	0.38	0.2	–	0.37	–	–	–	–	–	–	–	–	–	0.92	–	–	–	–
Salsola collina	–	–	–	–	–	–	–	0.77	–	–	–	–	0.96	0.67	2.15	7.43	–	–
**Amaranthaceae**	**0.38**	**0.2**	–	**0.37**	–	–	–	**0.77**	–	–	–	–	**0.96**	**1.58**	**2.15**	**7.43**	–	–
Androsace mariae	–	–	–	–	2.07	0.58	–	–	–	–	–	–	–	–	–	–	–	–
**Primulaceae**	–	–	–	–	**2.07**	**0.58**	–	–	–	–	–	–	–	–	–	–	–	–
Iris lactea	–	–	–	–	0.41	0.58	–	–	–	–	0.67	0.58	0.57	–	–	–	–	–
**Iridaceae**	–	–	–	–	**0.41**	**0.58**	–	–	–	–	**0.67**	**0.58**	**0.57**	–	–	–	–	–
Stellera chamaejasme	–	–	0.33	–	–	–	3.65	–	0.4	–	–	–	–	–	–	–	0.7	–
**Thymelaeaceae**	–	–	**0.33**	–	–	–	**3.65**	–	**0.4**	–	–	–	–	–	–	–	**0.7**	–

Plant species and family	RD (%)																	
	HN		HS		GN		GS		QF		TL		SI		HD		WY	
	April	July	April	July	April	July	April	July	April	July	April	July	April	July	April	July	April	July
Ephedra monosperma	10.38	–	9.67	–	–	–	7.3	–	–	–	–	–	–	–	11.55	11.05	9.19	–
**Ephedraceae**	**10.38**	–	**9.67**	–	–	–	**7.3**	–	–	–	–	–	–	–	**11.55**	**11.05**	**9.19**	–
Galium verum	–	–		0.98	–	–	–	–	–	–	–	–	–	–	–	–	–	–
**Rubiaceae**	–	–		**0.98**	–	–	–	–	–	–	–	–	–	–	–	–	–	–
Silene gracilicaulis	–	–	–	–	–	–	–	–	–	–	–	1.01	–	–	–	–	–	–
**Caryophyllaceae**	–	–	–	–	–	–	–	–	–	–	–	**1.01**	–	–	–	–	–	–
Polygonum sibiricum	–	0.5	–	–	–	–	–	–	–	–	–	–	–	–	–	–	–	–
**Polygonaceae**	–	**0.5**	–	–	–	–	–	–	–	–	–	–	–	–	–	–	–	–
Reaumuria songarica	–	–	–	–	–	–	–	–	–	–	–	–	–	–	–	–	–	2.32
**Tamaricaceae**	–	–	–	–	–	–	–	–	–	–	–	–	–	–	–	–	–	**2.32**
Limonium aureum	–	–	–	–	–	–	–	–	–	0.61	–	–	–	–	–	–	–	–
**Plumbaginaceae**	–	–	–	–	–	–	–	–	–	**0.61**	–	–	–	–	–	–	–	–

"-" indicates that the plant species was absent from the diet samples of the corresponding population.

Table S3 Importance values (IV) of plants in nine Przewalski's gazelle populations' habitats in July

Family	Species	HN	HS	GN	GS	TL	QF	SI	HD	WY
Poaceae	*Agropyron cristatum*	0.078	0.079	0.052	0.061	0.069	0.107	0.040	0.070	0.069
	Poa pratensis	0.133	0.062	0.125	0.080	0.087	0.179	0.025	0.003	0.009
	Agropyron desertorum	0.001	0.044	0.029	0.083	0.032	0.010	0.101	0.005	0.035
	Stipa purpurea	0.017	0.041	0.077	0.014	0.046	0.013	0.007	0.015	0.016
	Leymus secalinus	0.018	0.022	0.006	0.038	0.004	0.011	0.017	0.006	-
	Neotrinia splendens	0.002	0.023	0.004	0.013	0.016	0.021	0.032	0.012	0.086
	Elymus nutans	0.040	0.074	-	0.003	-	0.032	-	-	-
	Orinus kokonorica	-	-	-	0.006	-	-	0.250	0.311	0.110
	Koeleria macrantha	0.009	-	–	–	–	–	–	–	–
	Stipa sareptana	-	0.002	–	–	–	–	–	–	–
Cyperaceae	*Kobresia humilis*	0.033	0.048	0.122	0.088	0.153	0.051	0.015	0.029	0.212
	Carex arcatica	0.056	0.010	0.060	0.040	0.043	0.009	0.012	0.039	0.097
	Kobresia capillifolia	–	–	–	–	–	0.002	-	-	-
	Kobresia pygmaea	–	–	–	–	–	0.001	-	-	-
Asteraceae	*Artemisia frigida*	0.038	0.034	0.069	0.095	0.090	0.037	0.057	0.076	0.032
	Aster altaicus	0.068	0.026	0.013	0.049	0.003	0.107	0.034	0.051	0.029
	Taraxacum mongolicum	0.029	0.021	0.002	0.004	0.002	0.020	0.002	-	-
	Artemisia waltonii	0.016	0.048	0.014	0.002	0.043	0.031	0.014	0.026	0.005
	Artemisia salsoloides	–	–	–	–	–	–	0.026	0.104	-
	Xanthopappus subacaulis	0.014	0.002	0.014	0.002	-	0.001	-	0.003	-
	Leontopodium nanum	0.028	-	0.004	-	-	-	-	0.004	-
	Artemisia gmelinii	-	0.017	0.002	0.002	-	-	-	-	-

Family	Species	HN	HS	GN	GS	TL	QF	SI	HD	WY
	Artemisia phaeolepis	–	–	0.003	–	–	–	–	–	–
	Sonchus wightianus	–	–	–	–	–	–	–	–	0.006
	Cirsium souliei	0.009	0.005	0.010	0.002	–	0.013	0.005		0.007
	Aster flaccidus	–	–	–	–	0.013	–	–	–	–
	Cirsium arvense	–	0.007	–	–	–	–	–	–	–
	Artemisia sieversiana	0.002	–	–	–	0.009	–	0.011	–	–
	Saussurea pulchra	–	–	–	–	–	–	0.008	–	–
	Youngia simulatrix	0.013	–	–	–	–	–	–	–	–
	Anaphalis lactea	0.003	0.007	–	–	–	–	–	–	–
	Ajania tenuifolia	0.004	0.003	0.001	0.011	–	0.002	–	–	–
	Astragalus polycladus	0.041	0.022	0.069	0.030	0.051	0.047	0.012	0.021	0.005
	Oxytropis stracheyana	0.001	0.015	0.033	0.033	0.033	0.023	0.009	0.010	0.007
	Thermopsis lanceolata	0.004	0.021	0.004	0.017	0.005	0.005	0.026	0.010	–
Fabaceae	*Oxytropis falcata*	0.001	–	0.001	0.009	0.016	–	0.006	0.006	–
	Oxytropis aciphylla	–	–	–	–	–	–	0.022	–	–
	Oxytropis ochrocephala	0.003	0.002	–	–	–	–	–	–	–
	Astragalus laxmannii	–	–	–	0.005	0.007	–	–	–	–
	Medicago archiducis-nicolai	0.001	–	–	–	–	–	–	–	–
	Sibbaldianthe bifurca	0.012	0.042	0.050	0.057	0.023	0.016	0.003	0.012	0.033
	Potentilla multifida	0.041	0.022	0.042	0.015	0.017	0.029	–	–	0.022
Rosaceae	*Sibbaldianthe adpressa*	0.013	0.008	0.040	0.075	0.015	0.009	0.002	0.012	0.093
	Argentina anserina	0.033	0.060	–	0.003	–	0.013	0.002	–	–
	Dasiphora fruticosa	–	–	–	–	–	–	–	–	0.002
Apiaceae	*Bupleurum pusillum*	0.023	0.001	0.035	0.007	0.019	0.023	–	–	–

Family	Species	HN	HS	GN	GS	TL	QF	SI	HD	WY
	Carum carvi	0.007	0.001	-	-	0.001	0.005	-	-	-
	Semenovia malcolmii	0.009	0.002	0.002	0.003	0.019	-	-	-	-
	Lagotis brachystachya	0.011	0.048	0.001	0.014	0.008	0.009	-	0.002	-
	Pedicularis alaschanica	0.003	0.008	0.007	0.005	0.007	-	0.007	0.010	-
Scrophulariaceae	*Pedicularis kansuensis*	0.000	0.004	-	-	-	-	-	-	-
	Lancea tibetica	0.041	0.010	0.001	0.002	-	0.002	-	-	-
	Euphrasia regelii	0.001	0.001	-	-	-	-	-	-	-
Liliaceae	*Allium przewalskianum*	0.010	0.025	0.021	0.046	0.003	0.020	0.066	-	0.029
	Dracocephalum heterophyllum	0.007	0.010	0.007	0.024	0.041	0.002	0.032	0.042	-
Labiatae	*Elsholtzia densa*	0.003	-	-	-	-	0.003	-	-	-
	Scutellaria baicalensis	0.003	-	-	-	-	-	-	-	-
	Salsola collina		-	-	0.006	-	-	0.102	0.013	-
	Salsola tragus	-	-	-	-	-	-	-	0.014	0.013
Amaranthaceae	*Chenopodium album*	-	0.002	-	0.002	0.002	-	-	-	0.013
	Halogeton glomeratus	-	-	-	-	0.021	-	0.004	-	0.022
	Krascheninnikovia arborescens	-	-	-	-	0.003	-	0.019	-	-
Plantaginaceae	*Plantago depressa*	0.015	0.024	-	0.004	0.002	0.041	-	-	0.002
Iridaceae	*Iris lactea*	0.008	-	0.013	-	0.027	0.001	0.012	-	-
Elaeagnaceae	*Hippophae tibetana*	0.014	0.007	-	-	-	0.011	-	-	-
Thymelaeaceae	*Stellera chamaejasme*	0.019	0.001	0.008	0.001	0.030	0.001	-	0.033	0.007
Caryophyllaceae	*Silene nepalensis*	0.002	0.006	-	-	0.026	-	-	0.004	-
Ephedraceae	*Ephedra monosperma*	-	-	-	-	-	-	-	0.018	0.003
Primulaceae	*Androsace mariae*	0.023	0.046	0.033	-	-	0.030	-	-	-

Family	Species	HN	HS	GN	GS	TL	QF	SI	HD	WY
Convolvulaceae	*Convolvulus ammannii*	-	-	0.001	0.013	-	0.027	-	-	-
Linaceae	*Linum perenne*	-	-	-	-	-	-	0.002	0.002	-
Cruciferae	*Braya humilis*	0.003	0.007	0.016	0.017	0.006	0.014	0.020	0.026	0.007
	Lepidium apetalum	0.003	0.003	0.001	-	-	0.009	-	-	0.005
	Thlaspi arvense	-	0.003	-	-	-	-	-	-	-
Tamaricaceae	*Reaumuria songarica*	-	-	-	-	-	-	-	-	0.009
Nitrariaceae	*Nitraria sibirica*	-	-	-	-	-	-	-	-	0.014
Orobanchaceae	*Orobanche coerulescens*	-	-	-	-	0.001	-	-	-	-
Boraginaceae	*Asperugo procumbens*	-	-	-	-	0.001	-	-	-	-
	Lappula consanguinea	0.004	0.003		0.001	-	0.001	-	0.010	
Rubiaceae	*Galium verum*	0.013	0.001		0.001	-	-	-	-	-
	Galium spurium	-	-	-	0.001	-	-	-	-	-
Plumbaginaceae	*Limonium aureum*	-	-	-	-	-	0.005	-	-	-
Gentianaceae	*Gentiana dahurica*	0.011	0.005	0.004	0.007	-	0.002	0.002	-	-
	Gentiana straminea	0.003	0.001	-	-	-	-	-	-	-
	Gentianopsis paludosa	-	0.005	-	-	-	-	-	-	-
	Gentiana squarrosa	-	0.002	0.006	0.006	0.001	0.002	-	-	-
Ranunculaceae	*Ranunculus tanguticus*	-	0.003	-	-	-	-	-	-	-
Orchidaceae	*Herminium monorchis*	0.001	-	-	-	-	-	-	-	-
Polygonaceae	*Knorringia sibirica*	0.005	-	-	-	-	-	-	-	-

"-" indicates that the plant species was absent from the habitat of the corresponding population.

Table S4. Seasonal ranking of priority forage taxa for Przewalski's gazelle across the Qinghai Lake Basin based on availability-corrected dietary selectivity

ID	Plant	April			July			Categories
		n	Median D	Rank	n	Median D	Rank	
1	*Agropyron cristatum*	9	0.4892	1	9	0.262965	4	Core
2	*Aster altaicus*	9	0.4646	2	9	-0.04698	11	Core
3	*Artemisia frigida*	9	0.3898	3	9	0.418654	1	Core
4	*Kobresia humilis*	9	0.291	4	9	-0.15035	15	Spring-specific
5	*Leymus secalinus*	4	0.5905	5	4	0.657147	8	Core
6	*Thermopsis lanceolata*	6	0.25965	6	7	0.334988	2	Summer-specific
7	*Allium przewalskianum*	7	0.1141	7	7	0.270824	5	Summer-specific
8	*Stellera chamaejasme*	4	0.48395	8	1	-0.75267	26	Indicator species
9	*Poa pratensis*	7	0.1619	9	6	0.217588	10	Core
10	*Astragalus polycladus*	9	-0.1173	10	9	0.313621	3	Summer-specific
11	*Neotrinia splendens*	5	0.3868	11	4	-0.13899	21	Spring-specific
12	*Agropyron desertorum*	4	0.4433	12	6	0.086579	12	Spring-specific
13	*Sibbaldianthe bifurca*	6	0.21325	13	8	0.124433	9	Summer-specific
14	*Taraxacum mongolicum*	4	0.163	14	5	0.704576	6	Summer-specific
15	*Hippophae tibetana*	2	0.7281	15	3	0.72438	14	Local
16	*Oxytropis stracheyana*	5	0.0592	16	7	0.064692	13	Summer-specific
17	*Potentilla multifida*	7	-0.1521	18	7	0.353171	7	Summer-specific
18	*Ephedra monosperma*	2	0.84735	17	1	0.72438	23	Local

ID	Plant	April			July			Categories
		n	Median D	Rank	n	Median D	Rank	
19	*Bupleurum pusillum*	3	0.2337	19	5	-0.08856	17	Summer-specific
20	*Stipa purpurea*	2	0.1113	20	5	0.227771	16	–
21	*Lagotis brachystachya*	2	0.0672	21	4	-0.12229	19	–
22	*Plantago asiatica*	2	-0.21105	22	2	0.474043	18	–
23	*Orinus kokonorica*	3	-0.3084	23	3	-0.22623	25	–
24	*Carex arcatica*	1	-0.1452	24	3	-0.78987	24	–
25	*Salsola collina*	2	-0.2994	25	3	0.125088	20	–
26	*Elymus nutans*	1	-0.6204	26	3	-0.30723	22	–
